# Mitigation of Oxidative Damage Caused by Salinity in the Halophyte *Crithmum maritimum* L. via Biostimulant-Enhanced Antioxidant Activity

**DOI:** 10.3390/plants14243788

**Published:** 2025-12-12

**Authors:** Anastasia Evripidis Giannakoula, Michalis K. Stefanakis, Charikleia Papaioannou, Stavroula Tsimpliaraki, Sofia Kaftantzi, Alexandra Niniraki, Athanasios Gertsis

**Affiliations:** 1 Laboratory of Plant Physiology, Department of Agriculture, International Hellenic University, Sindos, 57400 Thessaloniki, Greece; alexandraninirak@gmail.com; 2 Department of Chemistry, University of Crete, Voutes, 71003 Heraklion, Greece; michstefanakis@yahoo.gr; 3 Laboratory of Applied Genetics & Breeding, Department of Biology, University of Patras, Rion, 26504 Patras, Greece; xpapaioannou@upatras.gr; 4 Department of Sustainable Agriculture and Management, Division of the American Farm School, Perrotis College, 57001 Thessaloniki, Greece; stavroulatsimpliaraki@gmail.com (S.T.); sofia.kaftantzi@gmail.com (S.K.); agerts@afs.edu.gr (A.G.)

**Keywords:** halophyte, salt stress, oxidative stress response, essential oils, GC-MS

## Abstract

The xerophyte medicinal species *Crithmum maritimum* was investigated for its physiological and antioxidant responses under increasing salinity stress and foliar biostimulant application. At moderate salinity (10 dS/m sodium chloride NaCl), plant growth and photosynthetic activity were enhanced, whereas high salinity (20 dS/m) led to significant reductions in biomass, photosynthetic efficiency, and water use efficiency. Salinity-induced oxidative stress was confirmed by elevated levels of malondialdehyde (MDA) and hydrogen peroxide (H_2_O_2_), in upper plant tissues. Foliar application of two bioactive compounds—Aquamin and Cultisano—partially mitigated the detrimental effects of high salinity. Treated plants maintained higher photosynthetic parameters and exhibited improved antioxidant profile compared to untreated controls. Furthermore, GC–MS analysis of essential oils revealed that salinity altered the composition of key volatile components, notably increasing *γ*-terpinene and reducing *p*-cymene content. Biostimulant treatments counteracted these changes, enhancing terpene components such as *p*-cymene, and thymol-methyl-ether. Overall, the findings highlight the potential of biostimulants to enhance both salt tolerance as well as the phytochemical value of *C. maritimum*, suggesting promising applications in sustainable agriculture and high-value plant product development under saline conditions. It was concluded that the type of biostimulant significantly influenced the physiological and quality characteristics of sea fennel plants. Further study on this topic is proposed, aiming at the improvement of antioxidant activity, which is beneficial to human health.

## 1. Introduction

Soil salinization represents a major constraint in modern agriculture, posing a serious threat to crop productivity and global food security. With an estimated 20% of irrigated lands already affected by salinity—a figure expected to rise due to climate change—there is an increasing demand for sustainable crops capable of thriving under saline conditions [1,2,3]. *Crithmum maritimum* L. (Apiaceae family), commonly known as sea fennel, is a native of Mediterranean coastal habitats and has recently emerged as a promising candidate for cultivation in saline environments due to its exceptional salt tolerance and high nutritional value [4,5,6]. Due to its natural salt tolerance, this species possesses significant economic and medicinal potential [7]. In addition, its adaptability to harsh coastal environments highlights its potential role in climate-resilient agricultural systems, where crop diversification is increasingly recognized as a key strategy for ensuring food security [8].

Halophytes are known to accumulate large amounts of bioactive metabolites under stressful conditions [9,10]. Moreover, the seeds of *C. maritimum* (rock samphire), a halophytic species native to coastal Mediterranean habitats and capable of completing its life cycle under a broad range of salinity regimes, contain a considerable amount of oil with a fatty acid composition similar to olive oil, making it a potential edible and industrial resource. Its leaves are rich in various compounds, including vitamin C, carotenoids, polyphenols, flavonoids, and other bioactive substances, with aromatic, medicinal, antimicrobial, and insecticidal applications [11,12]. The plant’s secondary metabolites (PSMs) play a vital role in its defense against a wide range of biotic and abiotic stresses, including salinity [13,14]. Recent studies suggest that sea fennel exhibits a dynamic metabolic plasticity, modulating both primary and secondary metabolism in response to environmental stressors, which underscores its value as a model species for stress physiology research [15].

Nevertheless, high salinity still imposes considerable physiological and biochemical stress on this plant, resulting in reduced growth and pigment content at elevated NaCl concentrations. Under stress, plants often synthesize in defense secondary metabolites with pharmaceutical potential [1,13]. To mitigate oxidative stress through non-enzymatic defense mechanisms, plants tend to accumulate PSMs under adverse conditions. Consequently, while salinity can decrease plant growth and yield, it may also stimulate the production of a diverse range of bioactive compounds. This dual response—growth inhibition coupled with enhanced metabolite biosynthesis—renders halophytes particularly attractive for the production of high-value phytochemicals, especially in low-input agricultural systems.

Recent research emphasizes the role of biostimulant—substances or microorganisms that enhance plant growth, nutrient uptake, and tolerance to abiotic stresses, thus improving plant resilience [16]. To enhance plant tolerance under suboptimal environmental conditions, the application of biostimulants—alongside complementary strategies such as conventional breeding and genetic engineering—is regarded as a promising approach [17]. Biostimulants function as supplementary inputs to fertilizers, with the primary objective of improving nutrient-use efficiency and enabling reductions in overall nutrient application rates.

When applied to plants, seeds, or soil, biostimulants, which include a broad range of natural compounds and beneficial microbes, promote growth and development by stimulating the plant’s intrinsic physiological processes, rather than acting as fertilizers [18,19]. Certain classes of biostimulants, such as seaweed extracts, humic substances, protein hydrolysates, and plant-growth-promoting rhizobacteria, have been shown to enhance antioxidant enzyme activity, osmotic adjustment, and ion homeostasis under saline conditions. Their eco-friendly nature and compatibility with sustainable agricultural practices make them promising alternatives to conventional chemical inputs [20].

This study investigates the effects of biostimulant treatments on *C. maritimum* under high salinity conditions. By evaluating physiological responses, antioxidant activity, and essential oil composition, we intend to provide evidence that biostimulants can mitigate the adverse effects of salt stress and enhance plant resilience. Furthermore, by analyzing the extracts and the chemical profile of its essential oils, we seek to identify the major bioactive constituents responsible for its distinctive aroma and/or therapeutic properties and to compare our data with those in previous studies. The results of this work may contribute to the development of sustainable cultivation strategies for sea fennel cultivation in marginal and saline soils.

## 2. Results

### 2.1. CO_2_ Assimilation Rate

**Figure 1:**
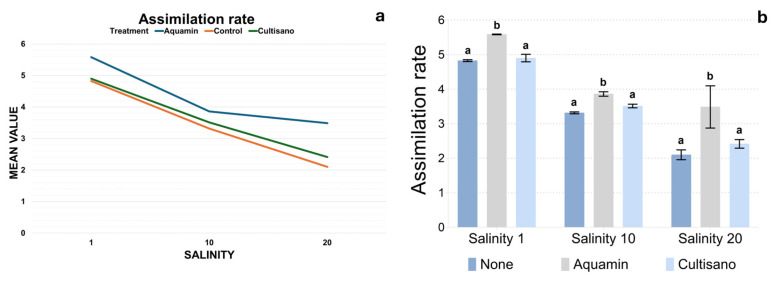
CO_2_ assimilation rate under control, salinity and biostimulant treatments. (**a**) Linear plot showing the mean assimilation rate (±SE) across salinity levels (1, 10 and 20 dS m^−1^). (**b**) Bar plots presenting treatment comparisons within each salinity level, with Tukey’s HSD groupings indicated by different letters.

The CO_2_ assimilation rate exhibited clear treatment-dependent responses across salinity levels (Figure 1a,b). At salinity 1 dS m^−1^, control plants showed values around 4.8 µmol CO_2_ m^−2^ s^−1^, whereas Aquamin-treated plants showed higher assimilation. Cultisano-treated plants displayed values close to the control but with slightly higher variability. Increasing salinity resulted in a progressive reduction in assimilation in all treatments, most noticeable in the control group, which dropped to ca. 3.3 µmol CO_2_ m^−2^ s^−1^ at salinity 10 dS m^−1^ and to 2.0–2.3 µmol CO_2_ m^−2^ s^−1^ at salinity 20 dS m^−1^. In contrast, Aquamin-treated plants maintained substantially higher values across salinity levels (3.68–3.98 at moderate salinity and 2.7 up to 4.19 at high salinity) while Cultisano treatments gave intermediate values. According to Tukey’s post hoc test, Aquamin-treated plants consistently belonged to a separate statistical group either the control and Cultisano-treated, across all salinity levels, indicating a stronger effect on CO_2_ assimilation. In contrast, Cultisano did not differ significantly from the control (Figure 1b). Mean values for all parameters are summarized in Supplementary Table S1.

### 2.2. CO_2_ Transpiration Rate

**Figure 2:**
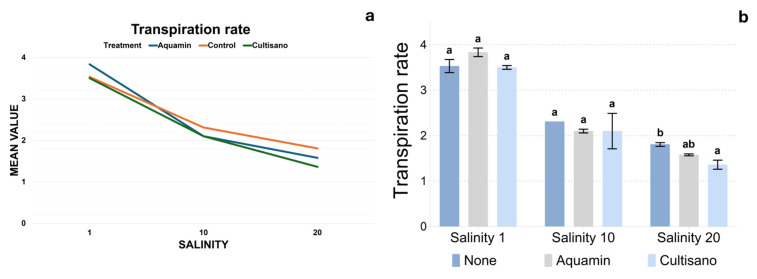
CO_2_ transpiration rate across treatments and salinity levels. (**a**) Linear plot illustrating the mean transpiration rate across salinity levels. (**b**) Bar plots showing mean transpiration rate (±SE) and Tukey’s HSD groupings indicated by different letters above the bars, show statistically significant differences between treatments within each salinity level.

Transpiration followed an overall similar pattern to assimilation (Figure 2a,b). At salinity 1 dS m^−1^, control plants exhibited transpiration values between 3.3 and 3.9 mmol H_2_O m^−2^ s^−1^, while Aquamin-treated plants displayed slightly higher rates (3.60–4.06). As salinity increased, the control group showed a uniform reduction (2.31 at moderate salinity; 1.74–1.92 at high salinity). Biostimulant treatments also showed a decline in transpiration values across salinity levels. Tukey’s test revealed no treatment differences at control and moderate salinities. At high salinity, the control formed a distinct lower subset, whereas Cultisano and Aquamin grouped into higher and intermediate subsets, respectively.

**Figure 3:**
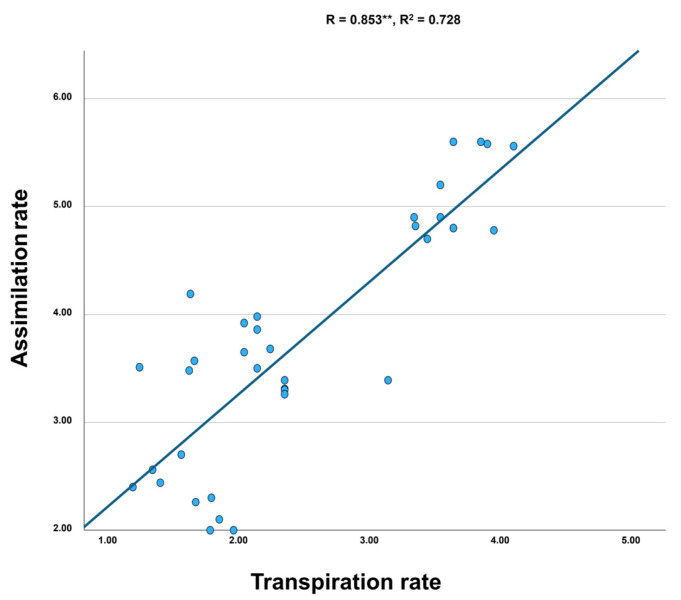
Scatter plot of CO_2_ assimilation versus CO_2_ transpiration across all treatments, showing the Pearson correlation coefficient (R) and coefficient of determination (R^2^) displayed on the plot. Double asterisks (**) indicate statistically significant correlations (*p* < 0.01).

A strong positive correlation between assimilation and transpiration (Figure 3) was supported by high Pearson R and R^2^ values. Summary statistics for all physiological measurements are provided in Supplementary Table S1.

### 2.3. GC–MS Volatile Composition

**Figure 4:**
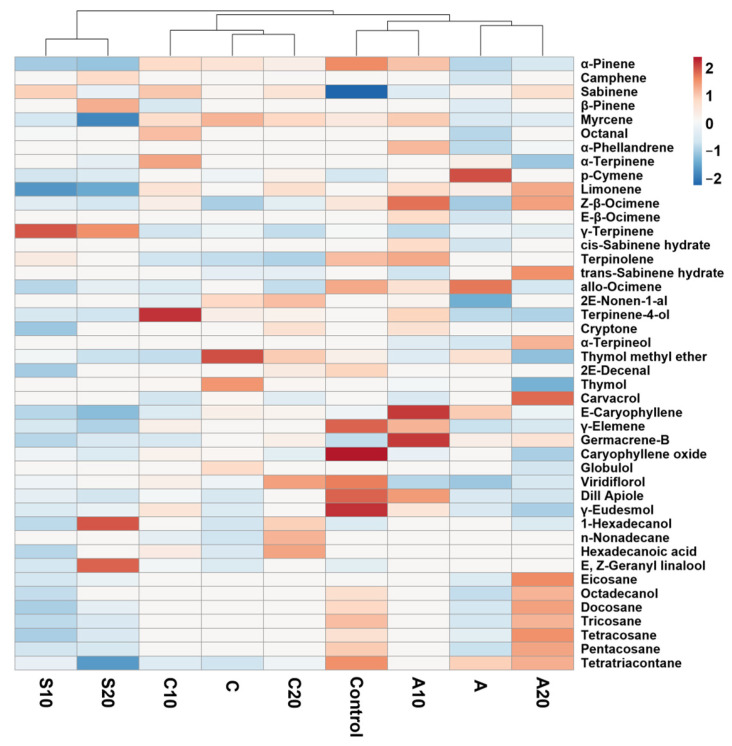
Heatmap of volatile compound abundances detected by GC–MS analysis across all treatments. Compounds and samples have been prioritized using hierarchical clustering based on Euclidean distance and Ward’s linkage method. Nonanal, trans-Limonene oxide and Tridecanol values were constant and were removed from the analysis.

GC–MS analysis revealed pronounced treatment-dependent shifts in volatile composition (Figure 4). For example, salinity increased several aliphatic constituents, with 1-hexadecanol reaching its highest levels in S20, while tetratriacontane declined markedly under salinity, reaching its lowest levels in S20. Several biostimulant treatments, particularly Cultisano, also exhibited reduced volatile levels relative to control. Monoterpene hydrocarbons presented distinct patterns: *γ*-terpinene and sabinene were elevated at both S10 and S20, whereas limonene reached its highest abundance in Aquamin-treated plants. Oxygenated monoterpenes, particularly terpinene-4-ol, thymol, and thymol methyl ether, varied distinctly, showing consistently higher values in the biostimulant treatments, especially in Cultisano. Sesquiterpene markers such as caryophyllene oxide, germacrene-B and E-caryophyllene also revealed pronounced treatment-dependent changes. The complete quantitative dataset of all identified volatile compounds, including retention times, chemical classes, retention indices, and relative abundances across treatments, is provided in Supplementary Table S2. These compound-level data underpin the heatmap clustering and subsequent multivariate analyses.

### 2.4. Oxidative Stress-Related VOC Analysis

**Figure 5:**
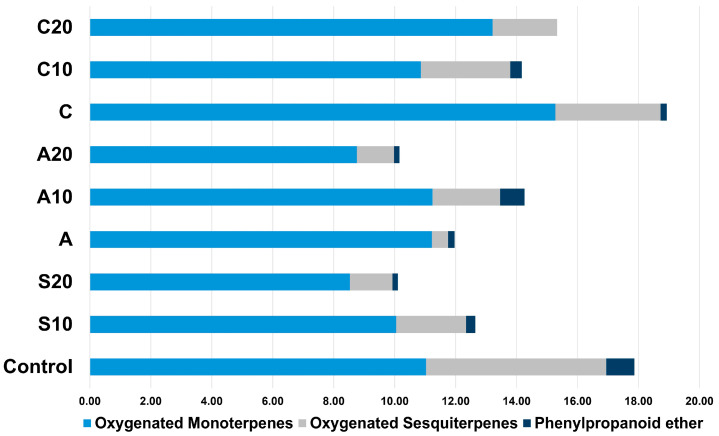
Stacked bar plots showing the relative proportions of oxidative stress-related volatile compound classes (oxygenated monoterpenes, oxygenated sesquiterpenes, and phenylpropanoid ether) across control, salinity (S10, S20), and biostimulant treatments (Aquamin: A, A10, A20; Cultisano: C, C10, C20). Values represent normalized relative abundances from GC–MS analysis.

The stacked bar plots of oxidative stress-related VOC classes (Figure 5) showed that salinity (S10, S20) led to a progressive reduction in oxygenated monoterpenes, oxygenated sesquiterpenes, and phenylpropanoid ether compared with the control. Biostimulant treatments displayed the opposite trend, with Aquamin maintaining class proportions close to control, while Cultisano produced the highest enrichment in oxygenated monoterpenes and moderate increases in oxygenated sesquiterpenes across salinity levels. Overall, salinity suppressed the synthesis of oxygenated VOC categories, while biostimulant application, especially Cultisano, resulted in higher overall proportions of oxygenated VOCs.

### 2.5. PCA of Volatile Profiles

**Figure 6:**
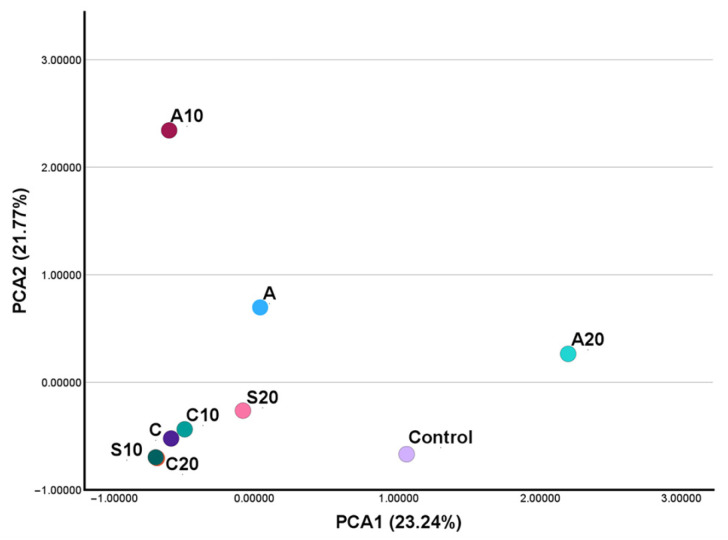
Principal Component Analysis (PCA) of volatile profiles across all treatments. The plot displays sample distribution along PC1 and PC2, which together explain 45.01% of total variance.

The PCA plot (Figure 6) showed clear differences among treatments. The salinity samples (S10, S20) grouped closely together in the lower left part of the plot, indicating similar VOC profiles. The control sample was positioned separately toward the right, showing an intermediate composition. Aquamin treatments were more spread out: A10 appeared higher on PC2, A was located near the center, and A20 shifted strongly to the right along PC1. Cultisano treatments formed a small cluster near the salinity samples, but were slightly higher on PC2. Overall, PC1 (23.24%) and PC2 (21.77%) captured most of the variation and separated the salinity, control, Aquamin and Cultisano groups.

### 2.6. Malondialdehyde (MDA)

**Figure 7:**
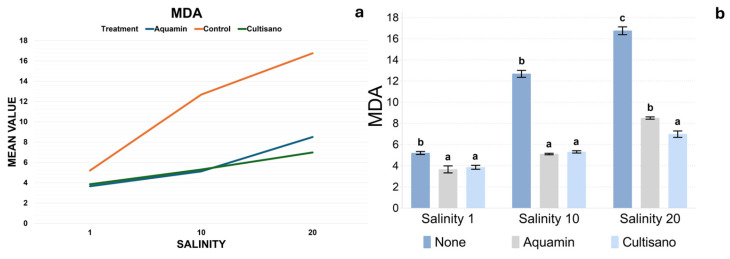
Malondialdehyde (MDA) concentration across treatments and salinity levels. (**a**) Linear plot showing MDA trends across salinity levels. (**b**) Bar plots showing mean MDA concentration (±SE) with Tukey’s HSD groupings. Different letters above the bars indicate statistically significant differences between treatments within each salinity level (*p* < 0.05).

MDA concentrations showed a strong salinity-dependent increase, especially in the control plants (Figure 7a,b). At salinity 1 dS m^−1^, control plants exhibited values of 5.0–5.6 nmol g^−1^ FW, whereas biostimulant-treated plants showed markedly lower MDA levels. At salinity 10 dS m^−1^, MDA increased highly in the control (11.9–13.5), while both Aquamin and Cultisano maintained significantly lower values. At salinity 20 dS m^−1^, the control reached the highest values in the dataset (15.8–17.6), while the biostimulant treatments retained substantially lower levels (6.4–8.7). Tukey’s test showed that at salinity 1 and 10 dS m^−1^, the control had higher MDA than both Aquamin and Cultisano values, which are no different from each other. At salinity 20 dS m^−1^, all treatments were separated statistically, with Cultisano having the lowest MDA, Aquamin intermediate levels, and the control having the highest. Mean values are summarized inSupplementary Table S1.

### 2.7. Total Phenolic Content (TPC)

**Figure 8:**
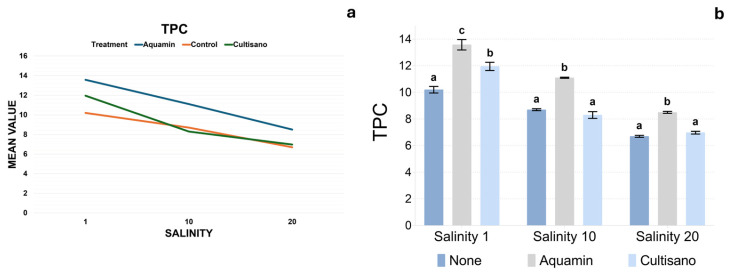
Total phenolic content (TPC) across treatments and salinity levels. (**a**) Linear plot showing TPC trends across salinity levels. (**b**) Bar plots showing mean TPC (±SE) with Tukey’s HSD groupings. Different letters above the bars indicate statistically significant differences between treatments within each salinity level (*p* < 0.05).

TPC displayed an opposite pattern to MDA (Figure 8a,b). At 1 dS m^−1^, TPC ranged between 9.6–10.8 mg GAE g^−1^ DW in control plants and was significantly higher under Aquamin (12.7–14.6) and Cultisano (11.1–12.5). Increasing salinity resulted in reductions in TPC in the control samples (6.6–8.8), whereas Aquamin maintained elevated values across salinity gradients. According to Tukey’s HSD, all treatments differed significantly at control salinity, with Aquamin showing the highest TPC, Cultisano intermediate values, and the control the lowest. At moderate and high salinity, Aquamin again formed a separate higher statistical group, while the control and Cultisano did not differ from each other. A full summary of mean TPC values for all treatments appears in Supplementary Table S1.

### 2.8. Correlation Analysis

**Figure 9:**
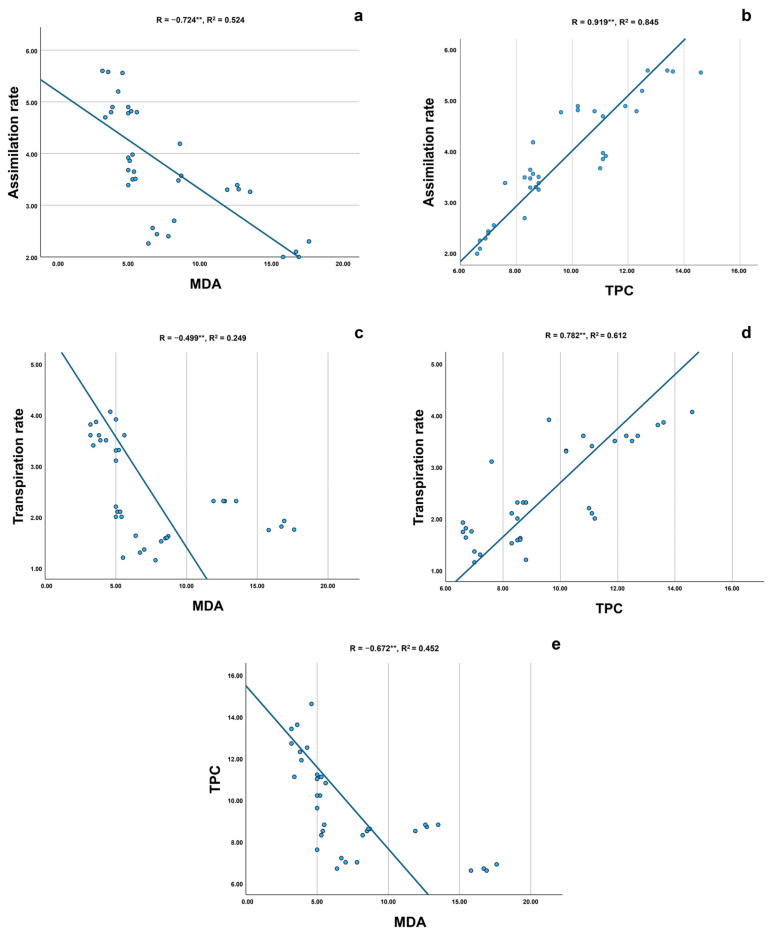
Correlation plots among physiological and biochemical parameters. (**a**) Scatter plot of CO_2_ assimilation rate vs. MDA concentration, showing Pearson correlation coefficient (R) and coefficient of determination (R^2^). (**b**) Scatter plot of CO_2_ assimilation rate vs. total phenolic content (TPC), with R and R^2^ displayed. (**c**) Scatter plot of CO_2_ transpiration rate vs. MDA concentration, with R and R^2^ displayed. (**d**) Scatter plot of CO_2_ transpiration rate vs. total phenolic content (TPC), with R and R^2^ displayed. (**e**) Scatter plot of MDA vs. total phenolic content (TPC), with R and R^2^ displayed. Double asterisks (**) indicate statistically significant correlations (*p* < 0.01).

Scatter plot analyses established strong and consistent relationships among physiological and biochemical parameters (Figure 9a–e). CO_2_ assimilation exhibited a strong positive correlation with both transpiration and TPC (Pearson R > 0.7), and a strong negative correlation with MDA. Transpiration followed similar patterns, showing positive associations with TPC and negative associations with MDA. TPC and MDA showed a clear inverse relationship across all treatments, supported by high R^2^ values that indicate a strong and consistent correlation.

## 3. Discussion

Salinity stress exerts profound effects on both the physiological performance and secondary metabolism of *C. maritimum* L. In this study, moderate salinity stimulated the biosynthesis of several stress-related monoterpenes, reflecting an adaptive metabolic adjustment, whereas higher salinity levels markedly inhibited photosynthetic activity and secondary metabolite production. These trends align with previous studies [1,21], which report that salinity can initially activate defensive metabolic pathways before exceeding the plant’s tolerance capacity and suppressing enzymatic and photosynthetic functions.

The application of biostimulants, particularly Aquamin and Cultisano, significantly enhanced the resilience of *C. maritimum* under saline conditions. Both products improved physiological performance by reinforcing antioxidant defenses, reducing lipid peroxidation, and maintaining pigment and metabolite stability. Aquamin, a marine-derived formulation rich in calcium and trace elements, likely stimulated enzymatic antioxidant pathways, contributing to the higher CO_2_ assimilation and transpiration rates observed across salinity levels. In contrast, Cultisano, which contains organic acids and micronutrients, appeared to support chlorophyll retention and strongly promoted the biosynthesis of oxygenated monoterpenes and other essential oil constituents. Similar biostimulant-mediated improvements in stress tolerance have been reported by other researchers [16,17,18].

The physiological responses observed in this study clearly show that salinity levels exceeding 10 dS m^−1^ markedly impaired plant growth, photosynthetic performance, and biomass accumulation in *C. maritimum.* Both CO_2_ assimilation and transpiration decreased significantly under increasing salinity, reflecting typical salt-induced restrictions in gas exchange. Such reductions are consistent with stomatal closure, declines in chlorophyll content, and potential inhibition of key photosynthetic enzymes. However, the application of Aquamin and Cultisano partially mitigated these effects, with Aquamin consistently sustaining the highest assimilation and transpiration rates across all salinity levels. These results suggest that the biostimulants enhance photosynthetic efficiency and carbon fixation under stress, likely through improved nutrient availability, strengthening of antioxidant defenses, and greater stabilization of chloroplast membranes as other researchers have previously reported [19].

These findings are consistent with previous reports indicating that elevated salinity disrupts the ionic and osmotic balance of *C. maritimum*, leading to impaired physiological performance [1]. In the present study, foliar application of Aquamin and Cultisano effectively mitigated these adverse effects, particularly at 10 dS m^−1^, where both biostimulants enhanced antioxidant status, stabilized physiological traits, and reduced visible stress symptoms. Aquamin appeared to strengthen antioxidant capacity, potentially through stimulation of enzymatic defense pathways, whereas Cultisano contributed to greater pigment retention and overall metabolic stability. Even at 20 dS m^−1^, biostimulant-treated plants maintained significantly higher chlorophyll content and biomass than untreated controls. These outcomes corroborate previous evidence that biostimulants enhance salinity tolerance by modulating antioxidant activity and improving nutrient uptake efficiency [22,23].

The recovery of *p*-cymene and terpinen-4-ol under biostimulant application suggests that these formulations not only alleviate salinity-induced oxidative stress but may also promote the biosynthesis of key bioactive metabolites [24]. Such compounds contribute to plant stress adaptation and enhance the medicinal and nutraceutical value of *C. maritimum*, highlighting the agronomic and economic relevance of incorporating biostimulants in halophyte cultivation.

Salinity stress and biostimulant treatments also induced pronounced modifications in the essential oil composition of *C. maritimum* leaves. Under control conditions, *p*-cymene (28.5%) and *γ*-terpinene (21.2%) were the dominant constituents. Exposure to salinity (10–20 dS m^−1^) significantly altered this profile. At 10 dS m^−1^, *γ*-terpinene increased to 37.1% while *p*-cymene declined to 16.9%, indicating a shift in monoterpene biosynthesis under moderate stress. At 20 dS m^−1^, *γ*-terpinene remained elevated (33.5%), whereas β-pinene and terpinen-4-ol declined sharply, reflecting the suppressive effects of severe salinity on secondary metabolism, in agreement with previous studies [24,25].

Biostimulant application mitigated these alterations to varying extents. At 20 dS m^−1^, Aquamin restored *p*-cymene levels to 36.4%, exceeding those of the control, while maintaining moderate concentrations of *γ*-terpinene (18.1%) and α-pinene (4.7%). Similarly, Cultisano sustained elevated *p*-cymene levels (32.5%) and markedly increased thymol-methyl-ether (11.2%), surpassing both control and salt-only treatments. Notably, Cultisano at 10 dS m^−1^ produced the highest terpinen-4-ol content (2.9%), a compound with recognized antimicrobial activity. These results indicate that biostimulants not only stabilize volatile biosynthesis under salt stress but may also selectively enhance the accumulation of valuable terpenoids, offering added potential for nutraceutical and essential oil applications. These findings align with previous reports showing that biostimulants can enhance antioxidant activity and stimulate secondary metabolism [26,27,28].

Compounds such as *β*-pinene and caryophyllene oxide, which declined markedly under salinity, were partially restored in plants treated with Aquamin or Cultisano, suggesting that these biostimulants exert a protective influence on terpene metabolism, potentially through modulation of enzymatic activity or gene expression. The detection of tetratriacontane (1.2%) in control plants—reduced under salinity and variably expressed following biostimulant application—further supports the notion that biostimulants can influence cuticular and defensive metabolic pathways under saline stress.

Total phenolic content (TPC) decreased progressively with increasing salinity, indicating that salt stress negatively affects either the biosynthesis or stability of phenolic compounds [25,29]. High salinity likely disrupts key metabolic pathways involved in secondary metabolite production, leading to reduced accumulation of antioxidant molecules. Similar declines in phenolics, terpenes and essential oil components under severe salinity have been reported in other aromatic species [30,31,32,33].

Both biostimulant treatments mitigated the reduction in TPC, with Aquamin showing the strongest positive effect across all salinity levels. The consistently higher TPC in Aquamin-treated plants suggests a stimulatory role in phenolic metabolism, potentially due to its high content of trace minerals and bioactive constituents that may enhance antioxidant enzyme activity and promote phenolic biosynthesis. Cultisano also improved phenolic retention relative to the control, albeit to a lesser extent, indicating a moderate protective effect.

The observed decline in phenolic content under salinity may reflect the diversion of metabolic energy toward essential survival processes and osmotic adjustment rather than secondary metabolite production. Nevertheless, the elevated phenolic levels maintained in both Aquamin- and Cultisano-treated plants indicate that these supplements may support antioxidant metabolism by enhancing redox balance or upregulating key enzymes in phenolic biosynthesis [34,35]. This interpretation is consistent with previous reports demonstrating that mineral-based products and biostimulants can increase stress tolerance through the stimulation of antioxidant pathways and the accumulation of phenolic compounds [19,20,34,35,36,37,38,39].

Overall, the results highlight the potential of targeted biostimulant applications as a sustainable agronomic strategy to alleviate salinity stress in halophytic crops such as *C. maritimum*. By partly restoring essential oil composition, enhancing phenolic metabolism, and improving physiological performance, Aquamin and Cultisano contribute to superior stress resilience. Nevertheless, further molecular and enzymatic studies are needed to elucidate the underlying mechanisms and to optimize application strategies for large-scale cultivation.

## 4. Materials and Methods

### 4.1. Plant Material and Experimental Design

The experiment was conducted at Perrotis College (Northern Greece; 40°34′ N, 22°58′ E) greenhouse facilities of the American Farm School in Thessaloniki, Greece and the biochemical analysis took place in the Laboratory of Plant Physiology of International Hellenic University in Sindos, Thessaloniki, Greece. One-year-old self-rooted *Crithmum maritimum* plants were grown in a greenhouse in 14-L pots filled with sandy-loam soil collected from the experimental site (65.2% sand, 29.6% silt, 15.2% clay; field capacity 2%). Inside the glasshouse, mean temperature ranged from 25 to 32 °C, relative humidity from 50 to 65%, and the photosynthetic photon flux density (PPFD) reached approximately 1200 µmol m^−2^ s^−1^ at midday on clear days.

A total of 45 black PVC pots (15 L each) were used, filled with a substrate (consisting of sand, silt, clay, and a small proportion of soil <2% *v*/*v*). The design followed a completely randomized block layout with two factors: salinity level and biostimulant treatment.

**AQUAMIN**, a product of Biosolids, is a water-soluble powder containing 62% plant-derived peptides. It enhances nutrient uptake and use efficiency while remaining a fully organic product.

**CULTISANO** contains chitosan (poly-D-glucosamine), a natural polymer derived from crustaceans, combined with amino acids obtained from enzymatic hydrolysis of plant proteins (Cultifort ).

For foliar application, **AQUAMIN** was diluted in water according to the manufacturer’s instructions (7 g L^−1^). For each application, 750 mL per pot was manually sprayed using a 1-L plastic hand sprayer.

Similarly, **CULTISANO** was diluted in water following the recommended dose (2 mL L^−1^) and applied foliarly in the same manner, with 750 mL sprayed per pot using a 1-L hand sprayer.

Three salinity levels were applied via irrigation water:

- 1 dS/m (control salinity);
- 10 dS/m (moderate salinity);
- 20 dS/m (high salinity).

At each salinity level, three biostimulant treatments were established:

1. Control (no biostimulant);
2. Foliar application of Aquamin;
3. Foliar application of Cultisano.

Each treatment was replicated five times, resulting in 15 pots per salinity level and a total of 45 pots. The pots were arranged in rows, with each row corresponding to a different salinity treatment. Within each row, the first five pots served as untreated controls, the next five received Aquamin, and the final five received Cultisano. The biostimulants were applied foliarly according to manufacturer instructions at regular intervals throughout the experimental period. Plants were grown under controlled greenhouse conditions. Data were collected on physiological and biochemical parameters, including biomass, pigment content, antioxidant activity, and stress indicators, following standard protocols.

### 4.2. Leaf Gas Exchange Measurements

A LI-COR 6410-XT portable infrared CO_2_ analyzer (LI-COR Biosciences, Lincoln, NE, USA) was used to measure photosynthetic assimilation rate (A) and transpiration rate (E), on fully expanded leaves in both differential and open circuit modes. Sixty days after the experiment began, all measurements were made in sunny conditions at 22 °C. The CO_2_ concentration inside the chamber was set at 400 μmol mol^−1^, and the photon flux density was 1000 μmol m^−2^ s^−1^. All photosynthetic parameters were calculated following Von Caemmerer and Farquhar [40].

### 4.3. Total Polyphenol Content (TPC)

The TPC was evaluated according to a previous methodology, Giannakoula et al. [39], with some modifications [41]. An Eppendorf tube was used to mix 100 μL of Folin–Ciocalteu reagent with 100 μL of a sample extract. After 2 min, 800 μL of 5% *w*/*v* Na_2_CO_3_ solution was added. After vortexing, the mixture was incubated for 30 min at room temperature. The absorbance at 760 nm was measured using a Shimadzu UV-1700 PharmaSpec Spectrophotometer from Kyoto, Japan. The results were expressed in gallic acid equivalents per gram of dry weight (GAE; mg/g DW) using a gallic acid standard curve.

### 4.4. GC–MS Analysis of Volatile Oils

GC–MS analysis of the isolated volatile oils was performed on a Shimadzu GC-17A gas chromatograph coupled with a Shimadzu GCMS-QP 5050 mass-selective Quadrupole Mass Spectrometer as a detector with the appropriate data system (Shimadzu Corp., Kyoto, Japan). The GC was equipped with a Grob-type split-splitless injector, the fused silica capillary column (Supelco, Bellefonte, PA, USA, SBP-5 with 0.25 μm film thickness, 30 m × 0.25 mm i.d.) was directly coupled to the ion source. Helium was used as a carrier gas with a back pressure of 0.8 Atm. The injector temperature was 250 °C, and the oven temperature program started at 50 °C for 5 min and then increased at a rate of 5 °C/min up to 150 °C, was retained at this temperature for 10 min, and increased again at a rate of 5 °C/min up to 280 °C, where it remained for 20 min. The scanning range was 30–700 *m*/*z*. A GS-MS detection electron ionization system was used with an ionization energy of 70 eV.

#### Identification and Quantification of Volatile Components

The quantification of the components was based on the total number of fragments (total ion count) of the metabolites, as detected by the mass spectrometer. The identification of the chemical components was carried out based on the retention time of each component (Rt) compared with those of commercially available compounds, by analysis of their mass spectra, by the use of the NIST21, NIST107 and PMW_TOX2 mass spectra libraries [42], as well as by comparison with literature data [43]. Calculation of retention indices was performed in accordance with the work of [44], in comparison to the retention times of standard hydrocarbons (C_8_–C_40_).

### 4.5. Determination of H_2_O_2_ Concentration

For the determination of H_2_O_2_ concentration in leaves of *C. maritinum* L. plants after 60 days of biostimulants treatment, leaf extraction was carried out according to Giannakoula et al. [45]. Hydrogen peroxide was measured spectrophotometrically after reaction with KI. The reaction mixture consisted of 0.5 mL leaf extract with 0.1% trichloroacetic acid (TCA), 0.5 mL of 100 mM K-phosphate buffer and 2 mL reagent (1 M KI in fresh double-distilled H_2_O). The blank consisted of 0.1% TCA. The reaction was developed for 1 h in the dark. The absorbance of the solution was measured at 410 nm, and the H_2_O_2_ concentration was calculated using a standard curve ranging from 0.1 to 1 mm. H_2_O_2_ content and was expressed as nmol g^−1^ FW.

### 4.6. Malondialdehyde (MDA) Concentration

The level of lipid peroxidation of *C. maritinum* L. leaves at the end of the experiment was measured and the MDA content was determined by reaction with 2-thiobarbituric acid (TBA) according to [46]. Fresh leaf blade tissue, 0.1 g, was homogenised by adding 0.5 mL of 0.1% (*w*/*v*) trichloroacetic acid (TCA). The homogenate was centrifuged at 15,000× *g* and 4 °C for 10 min. From the supernatant, 0.5 mL was mixed with 1.5 mL of 0.5% TBA diluted in 20% TCA. Incubation follows at 95 °C for 25 min. The reaction stops by incubating in an ice bath. Afterward, the tubes were centrifuged at 10,000× *g* and 4 °C for 10 min and the absorbance of the supernatant was read at 532 and 600 nm. The value for the non-specific absorption at 600 nm was subtracted from the value at 532 nm. The concentration of MDA was calculated using Lambert–Beer’s law using the MDA extinction coefficient of 155 mM ^−1^ cm^−1^. Results are presented as nmol MDA g^−1^ FW.

### 4.7. Statistical Analysis

Statistical analyses were performed using SPSS v29 (IBM, Armonk, NY, USA), GraphPad Prism v8 (GraphPad Software, Boston, MA, USA), and Microsoft Excel. Essential oil composition data derived from GC–MS analysis were visualized in ClustVis [47] using z-score normalization and hierarchical clustering based on Euclidean distances and Ward’s method. Relative essential oil abundances, including key compounds associated with oxidative stress, were illustrated using stacked bar plots in Excel. Principal Component Analysis (PCA) was conducted in SPSS v29 to assess the separation among the nine treatment groups (Control, S10, S20; Aquamin: A, A10, A20; Cultisano: C, C10, C20) according to their volatile profiles.

Physiological and biochemical parameters (CO_2_ assimilation rate, CO_2_ transpiration rate, malondialdehyde—MDA concentration, and total phenolic content—TPC) were analyzed using two-way ANOVA, with Treatment categories (None, Aquamin, and Cultisano) and Salinity considered as fixed factors. When significant effects were detected, Tukey’s HSD post hoc test was applied to determine differences among means. Data were visualized using bar plots with standard error bars, while linear plots were used to depict trends across salinity levels. Correlations among physiological and biochemical variables were evaluated using Pearson correlation analysis, and the corresponding correlation coefficients (R) and coefficients of determination (R^2^) were reported [47].

## 5. Conclusions

In conclusion, salinity stress substantially affected the physiological performance, essential oil composition, and phenolic metabolism of *C. maritimum*. Increasing salinity levels, particularly above 10 dS m^−1^, impaired growth, reduced photosynthetic activity, and suppressed the biosynthesis of several key volatile compounds. The foliar application of Aquamin and Cultisano effectively mitigated these detrimental effects, with Aquamin generally providing the greatest improvement across physiological parameters, phenolic content, and monoterpene stability.

Both biostimulants enhanced plant resilience by improving pigment retention, supporting antioxidant capacity, and partially restoring salinity-suppressed terpenoids such as *p*-cymene, *β*-pinene, and terpinen-4-ol. Notably, Aquamin at 20 dS m^−1^ restored *p*-cymene to levels higher than the control, while Cultisano selectively increased compounds such as thymol-methyl-ether and terpinen-4-ol, indicating a stimulatory effect on specific branches of monoterpene biosynthesis. These responses highlight the dual action of biostimulants in alleviating oxidative stress while enriching valuable bioactive metabolites.

Overall, the findings demonstrate that targeted biostimulant applications can significantly enhance the tolerance of halophytic crops to saline environments by stabilizing physiological function and promoting the production of antioxidant and antimicrobial secondary metabolites. Such strategies offer promising potential for the sustainable cultivation and valorization of *C. maritimum* in salt-affected agricultural systems. Further molecular analyses are warranted to elucidate the underlying regulatory mechanisms and optimize application regimes for large-scale production.

## Data Availability

The data presented in this study are available on request from the corresponding author. The data are not publicly available due to privacy and ethical restrictions.

## Supplementary Materials

The following supporting information can be downloaded at https://www.mdpi.com/article/10.3390/plants14243788/s1. **Supplementary Table S1:** Mean values (±SD) of CO_2_ assimilation rate (µmol CO_2_ m^−2^ s^−1^), transpiration rate (mmol H_2_O m^−2^ s^−1^), malondialdehyde (MDA; nmol g^−1^ FW) and total phenolic content (TPC; mg GAE g^−1^ FW) measured in Crithmum maritimum plants across three salinity levels (1, 10 and 20 dS m^−1^) and three treatments (Control, Aquamin, Cultisano). Each value represents the mean of n = 5 plants per treatment. **Supplementary Table S2:** Volatile organic compounds (VOCs) identified by GC–MS analysis in *Crithmum maritimum* across control, salinity (S10, S20) and biostimulant treatments (Aquamin: A, A10, A20; Cultisano: C, C10, C20); R.I.^a^ (Retention Indices) from experimental using a SBP-5 column using a homologous series of n-alkanes (C8–C40); R.I.^b^ (Retention Indices) from literature data, Adams (2007) and National Institute of Standards and Technology (2025).
