# Supplementary material for: Mitigation of Oxidative Damage Caused by Salinity in the Halophyte Crithmum maritimum L. via Biostimulant-Enhanced Antioxidant Activity
Source: Plants (Basel). 2025 Dec 12;14(24):3788. doi: 10.3390/plants14243788 (PMC12737243; doi:10.3390/plants14243788)
Supplement: Supplementary file 1 [file plants-14-03788-s001.zip › plants-3992656-supplementary.pdf]

Mitigation of Oxidative Damage Caused by Salinity in the Halophyte *Crithmum maritimum* L. via Biostimulant-Enhanced Antioxidant Activity

Anastasia E. Giannakoula[1*], Michalis K. Stefanakis[2], Charikleia Papaioannou[3], Stavroula Tsimpliaraki[4], Sofia Kaftantzi[4], Alexandra Ninaraki[1] and Athanasios Gertsis[4]

[1]Laboratory of Plant Physiology, Department of Agriculture, International Hellenic University, Sindos, 54700, Greece (A.E.G.; agianna@ihu.gr (A.G.);
[2]Department of Chemistry, University of Crete, Voutes, 71003 Heraklion, Greece (M.K.S; michstefanakis@yahoo.gr)
[3]Laboratory of Applied Genetics & Breeding, Department of Biology, University of Patras, Rion, 26504, Greece xpapaioannou@upatras.gr
[4]Department of Sustainable Agriculture and Management, Perrotis College, Division of the American Farm School, Thessaloniki, Greece; stavroulatsimpliaraki@gmail.com (S.T.); sofia.kaftantzi@gmail.com (S.K.); alexandraninirak@gmail.com (A.N.); agerts@afs.edu.gr (A.Ge.).
*Correspondence: ;agianna@ihu.gr;

Supplementary Table S1. Mean values (±SD) of CO_2 assimilation rate (μmol CO_2 m^{-2} s^{-1}), transpiration rate (mmol H_2O m^{-2} s^{-1}), malondialdehyde (MDA; nmol g^{-1} FW) and total phenolic content (TPC; mg GAE g^{-1} FW) measured in *Crithmum maritimum* plants across three salinity levels (1, 10 and 20 dS m^{-1}) and three treatments (Control, Aquamin, Cultisano). Each value represents the mean of n = 5 plants per treatment.

Treatments	Assimilation Rate of CO_2	Transpiration Rate of CO_2	MDA	TPC
**Salinity 1**				
Control	4.83 ± 0.05	3.53 ± 0.29	5.20 ± 0.28	10.20 ± 0.49
Aquamin	5.59 ± 0.02	3.83 ± 0.66	3.65 ± 0.78	13.58 ± 0.78
Cultisano	4.90 ± 0.22	3.85 ± 0.37	11.95 ± 0.62	11.95 ± 0.62
**Salinity 10**				
Control	3.32 ± 0.05	2.31 ± 0.00	12.68 ± 0.66	8.70 ± 0.14
Aquamin	3.86 ± 0.13	2.10 ± 0.08	5.10 ± 0.14	11.10 ± 0.08
Cultisano	3.51 ± 0.11	2.10 ± 0.78	5.30 ± 0.22	8.30 ± 0.51
**Salinity 20**				
Control	2.10 ± 0.14	1.81 ± 0.08	16.75 ± 0.74	6.70 ± 0.14
Aquamin	3.49 ± 0.61	1.58 ± 0.04	8.50 ± 0.22	8.50 ± 0.14
Cultisano	2.42 ± 0.12	1.36 ± 0.20	6.98 ± 0.60	6.98 ± 0.21

Supplementary Table S2. Volatile organic compounds (VOCs) identified by GC–MS analysis in *Crithmum maritimum* across control, salinity (S10, S20) and biostimulant treatments (Aquamin: A, A10, A20; Cultisano: C, C10, C20); [a]R.I. (Retention Indices) from experimental using a SBP-5 column using a homologous series of n-alkanes (C8–C40); [b]R.I. (Retention Indices) from literature data, Adams (2007) and National Institute of Standards and Technology (2025).

No	Ret. Time	Compounds	R.I.[a]	R.I. [b]	Control	S10	S20	A	A10	A20	C	C10	C20
1	13.119	α-Pinene	939	939	6.76	4.23	4.14	4.39	6.24	4.70	5.79	5.99	5.53
2	14.237	Camphene	954	954			6.30	0.09					
3	17.378	Sabinene	975	975	13.13	20.73	17.98	18.98	17.53	20.17	18.96	21.01	19.93
4	17.955	β-Pinene	979	980			1.11	0.30				0.23	
5	18.288	Myrcene	990	990	2.29	1.38	0.31	1.47	2.72	1.55	2.92	2.53	2.60
6	18.983	Octanal	995	995		0.57		0.11				1.09	
7	19.606	α-Phellandrene	1002	1002				0.48	1.27	0.79			
8	19.814	α-Terpinene	1017	1016			0.43	0.54		0.28		0.74	
9	20.565	p-Cymene	1024	1024	0.37	0.31	0.61	4.68			1.20		1.71
10	20.900	Limonene	1029	1030	28.38	16.82	18.49	30.18	32.89	36.42	28.41	31.96	32.44
11	21.700	Z-β-Ocimene	1037	1037	1.55	1.26	1.18	1.05	1.96	1.84	1.07	1.49	1.28
12	22.403	E-β-Ocimene	1050	1050				0.06	0.20				
13	23.911	γ-Terpinene	1059	1059	21.21	37.10	33.52	20.10	13.70	18.02	19.72	15.36	14.42
14	24.791	E-Sabinene hydrate	1070	1070				0.06	0.14				
15	27.597	Terpinolene	1088	1088	2.24	1.67			2.41		0.68	0.77	0.58
16	28.934	Z-Sabinene hydrate	1098	1098					0.09	0.46	0.16		0.16
17	29.993	Nonanal	1100	1100								0.06	
18	33.674	allo-Ocimene	1132	1132	0.72	0.22	0.36	0.82	0.58	0.29		0.32	0.24
19	34.473	Z-Limonene oxide	1142	1140					0.11				
20	39.056	2E-Nonen-1-al	1161	1162				0.06	0.24		0.31	0.18	0.34
21	41.196	Terpinene-4-ol	1177	1177	1.28	0.80	0.69	0.57	1.89	0.50	1.47	2.87	1.40
22	41.539	Cryptone	1185	1185		0.12			0.15				0.15
23	42.388	α-Terpineol	1188	1188				0.11	0.12	0.18			

No	Ret. Time	Compounds	R.I.[a]	R.I. [b]	Control	S10	S20	A	A10	A20	C	C10	C20
24	45.724	Thymol methyl ether	1235	1235	9.75	9.13	7.84	10.48	8.44	7.05	13.10	7.70	11.17
25	46.874	2E-Decenal	1263	1262	0.31	0.07							0.25
26	48.055	Thymol	1290	1290					0.13	0.01	0.26	0.14	0.14
27	48.380	Carvacrol	1299	1298					0.17	0.56	0.28	0.15	0.19
28	49.445	E-Caryophyllene	1419	1420	0.51	0.26	0.16	0.85	1.21	0.50	0.63	0.39	0.60
29	52.557	γ-Elemene	1436	1434	0.66	0.17	0.09	0.13	0.53	0.17		0.34	0.30
30	54.918	Germacrene-B	1561	1562	1.07	1.02	1.26	1.80	2.95	1.98		1.23	1.77
31	55.890	Tridecanol	1571	1570				0.03	0.03				
32	57.451	Caryophyllene oxide	1583	1582	4.20	1.54	1.13		1.41	0.62	1.79	1.87	1.27
33	57.513	Globulol	1590	1590						0.06	0.91		
34	57.563	Viridiflorol	1592	1590	0.91	0.54		0.33	0.37	0.44	0.54	0.62	0.85
35	58.278	Dill Apiole	1620	1650	0.92	0.30	0.18	0.22	0.80	0.18	0.21	0.38	
36	58.634	γ-Eudesmol	1632	1632	0.80	0.21	0.27	0.20	0.44	0.10	0.21	0.44	
37	63.904	1-Hexadecanol	1875	1874	0.23	0.10	1.12			0.24	0.17	0.44	0.74
38	67.098	n-Nonadecane	1900	1900							0.20	0.30	0.64
39	67.424	Hexadecanoic acid	1960	1960		0.06					0.14	0.34	0.55
40	67.925	E, Z-Geranyl linalool	1987	1987	0.18	0.05	1.29				0.12	0.32	
41	69.921	Eicosane	2000	2000		0.02	0.07	0.04		0.27			
42	71.664	Octadecanol	2077	2077	0.23	0.03		0.03		0.30			
43	73.369	Docosane	2200	2200	0.18	0.02	0.08	0.05		0.23			
44	74.981	Tricosane	2300	2300	0.20	0.02	0.05	0.04		0.21			
45	76.531	Tetracosane	2400	2400	0.17	0.02	0.06	0.08		0.25			
46	78.032	Pentacosane	2500	2500	0.25	0.05	0.06	0.04		0.30			
47	81.225	Tetratriacontane	3400	3400	1.20	0.60	0.11	1.00	0.70	1.11	0.43	0.52	0.64
		**Total %**			**99.70**	**99.42**	**98.89**	**99.37**	**99.42**	**99.78**	**99.68**	**99.78**	**99.89**
